# The validity and reliability of the four square step test in different adult populations: a systematic review

**DOI:** 10.1186/s13643-017-0577-5

**Published:** 2017-09-11

**Authors:** Martha Moore, Karen Barker

**Affiliations:** Nuffield Orthopaedic Centre, Oxford University Hospitals NHS Foundation Trust, Oxford, OX3 7HE UK

**Keywords:** Systematic review, Outcome measures, Validity, Reliability

## Abstract

**Background:**

The four square step test (FSST) was first validated in healthy older adults to provide a measure of dynamic standing balance and mobility. The FSST has since been used in a variety of patient populations. The purpose of this systematic review is to determine the validity and reliability of the FSST in these different adult patient populations.

**Methods:**

The literature search was conducted to highlight all the studies that measured validity and reliability of the FSST. Six electronic databases were searched including AMED, CINAHL, MEDLINE, PEDro, Web of Science and Google Scholar. Grey literature was also searched for any documents relevant to the review. Two independent reviewers carried out study selection and quality assessment. The methodological quality was assessed using the QUADAS-2 tool, which is a validated tool for the quality assessment of diagnostic accuracy studies, and the COSMIN four-point checklist, which contains standards for evaluating reliability studies on the measurement properties of health instruments.

**Results:**

Fifteen studies were reviewed studying community-dwelling older adults, Parkinson’s disease, Huntington’s disease, multiple sclerosis, vestibular disorders, post stroke, post unilateral transtibial amputation, knee pain and hip osteoarthritis. Three of the studies were of moderate methodological quality scoring low in risk of bias and applicability for all domains in the QUADAS-2 tool. Three studies scored “fair” on the COSMIN four-point checklist for the reliability components. The concurrent validity of the FSST was measured in nine of the studies with moderate to strong correlations being found. Excellent Intraclass Correlation Coefficients were found between physiotherapists carrying out the tests (ICC = .99) with good to excellent test-retest reliability shown in nine of the studies (ICC = .73–.98).

**Conclusions:**

The FSST may be an effective and valid tool for measuring dynamic balance and a participants’ falls risk. It has been shown to have strong correlations with other measures of balance and mobility with good reliability shown in a number of populations. However, the quality of the papers reviewed was variable with key factors, such as sample size and test set up, needing to be addressed before the tool can be confidently used in these specified populations.

**Electronic supplementary material:**

The online version of this article (10.1186/s13643-017-0577-5) contains supplementary material, which is available to authorized users.

## Background

**Fig. 1:**
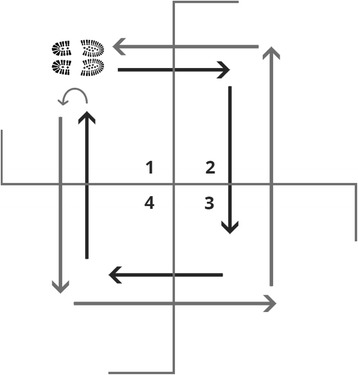
FSST setup: the test starts in square 1, facing square 2. The subject stays facing in this direction as they step into squares 2, 3, 4, 1, 4, 3, 2 and back to 1

Foot clearance is an important function required in everyday life. The ability to do this in different directions is essential when reacting to stimuli such as navigating crowds in a busy street or walking on uneven pavement. Compared to straight path walking, walking a curved path and changing direction further challenges balance and requires increased motor planning [1]. The four square step test (FSST) was developed by Dite and Temple [2] and incorporates rapid stepping whilst changing direction. The test requires a person to step forwards, backwards and sideways over obstacles in a specified sequence (see Fig. 1).

First validated in healthy older adults, the FSST has been shown to provide a measure of dynamic standing balance and mobility. Both balance and mobility are the most consistently identified risk factors linked to falls [3, 4], with one in three people over the age of 65 and half of those over the age of 80 falling once every year [5]. It is also understood due to the multifactorial nature of balance and mobility that not every aspect of the two can be incorporated into one single measurement tool. However, most falls occur during movement itself, with trips and slips making up a large proportion of falls [6–8], indicating that the ability to take a rapid step may help prevent some of these falls. This aspect of balance and mobility has received little attention in the past and so it seems pertinent now to identify and analyse if this measure is an accurate and reliable indicator of balance and falls risk.

The only other measure developed to test rapid step taking is the step test [9]. The participant is asked to step one foot on, then off, a set height block repeatedly as quickly as they can. They are given 15 s for each trial and both lower extremities are tested [10]. To date, the step test has only been studied in community-dwelling older adults and stroke patients [9–11], which has shown a good correlation between lower limb muscle strength, walking speed, lower limb coordination and balance [11]. Despite this, the step test only assesses rapid step taking in the forward direction when it is known that in an impending fall situation, stepping in different directions is required to maintain balance [12]. What is more, age has been found to decrease stepping speed in forward, backward and sideways directions [13] whilst also distinguishing between fallers and non-fallers [12]. Therefore, a test of just forward stepping will not provide an accurate measure of the patient’s functional mobility and balance.

Unlike the step test, the FSST involves the participant transferring their full weight between each leg whilst stepping at speed and in forward, backward and sideway directions over an obstacle [9]. The FSST is also more cognitively demanding, getting participants to remember a set sequence of steps and then complete it. Another key difference is the FSST allows the participant to use their walking aid whilst completing the test, compared to the step test which does not. These differences, however, can present both advantages and disadvantages depending on the testing population [2].

The FSST has also been noted to have some key disadvantages. These include the higher level of physical supervision required whilst the participant is carrying out the test and sometimes the need for a second assessor for participants who have greater balance impairments [2]. This presents a challenge in clinical scenarios, where pressure on therapist’s time is increased and two therapists cannot be present. Secondly, the FSST imposes a testing limit meaning if the person fails to complete the test more than twice for each trial, they will not be given a score [2]. This failure to award a score may produce a floor effect in those testing populations where there is a greater balance or cognitive impairment.

For healthcare professionals to be able to use the FSST clinically, it is important that the test is validated in the relevant populations and its reliability between testers gauged. Given that there are many factors that can cause balance and mobility problems from hearing impairments to long-term neurological conditions, many older adults and those with medical conditions will be at increased risk of falling. For effective rehabilitation to reduce this risk, the FSST will need to be validated in these populations so it can be known if the test is accurate. Many studies have looked at the validity and reliability of the FSST in a variety of patient populations since it was first devised in 2002. However, no previous reviews have been undertaken to provide an overview for clinicians wishing to utilise the FSST. To allow a thorough and in-depth analysis of the use and role of the FSST within the clinical setting, multiple patient populations were included in this review.

The objective of this systematic review is to, therefore, establish the validity and reliability of the FSST compared to other commonly used outcome measures for balance and mobility in different adult populations. The feasibility of the FSST in different testing populations will also be analysed.

## Methods

### Search strategy

We identified that there were insufficient reviews published to base our review on one type of research design alone. For example, RCTs and instead chose to adopt a comprehensive search of all studies that had explored the use of the FSST in a clinical population irrespective of study design [14]. A systematic literature search was performed in December 2015 and again updated in January 2017, which included the following databases: AMED, CINAHL, MEDLINE, SPORTDiscus and Web of Science. The search was further performed on one search engine, Google Scholar, and one supplementary database, the Physiotherapy Evidence Database (PEDro). Grey literature was searched using the NICE Health and Social Care Evidence search and opengrey.eu. No additional relevant documents were found through this route. Additionally, reference lists of key papers were crosschecked and a citation search was completed on Web of Science. A combination of keywords and MeSH terms were used as shown in Table 1.

**Table 1:** Search terms for databases and search engines

Database	Syntax
AMED	(“The four square step test” KW) AND (valid* KW) OR (reliab* KW) OR (sensitiv* KW) OR (reproducib* KW)
CINAHL	(“The four square step test” KW) AND (validation studies MH OR validation therapy MH) OR (reliability MH OR reliability AND validity MH) OR (sensitivity and specificity MH) OR (reproducibility of results MH)
MEDLINE	(“The four square step test” KW) AND (validation studies MH OR validation studies as topic MH) OR (reliab* KW) OR (sensitivity and specificity MH) OR (reproducibility of results MH)
Web of Science	(“The four square step test” TS) AND TITLE-ABS-KEY: (valid* TS) OR (reliab* TS) OR (sensitiv* TS) OR (reproducib* TS)
SPORTDiscus	(“The four square step test” KW) AND (valid* KW) OR (reliab* KW) OR (sensitiv* KW) OR (reproducib* KW)
PEDro	(“The four square step test”)
Google Scholar	(“The four square step test”)

*KW* keyword, *MH* MeSH heading, *TS* topic search

The search strategy was reviewed by an independent librarian and conducted following the Cochrane handbook search strategy guidelines [15]. The study was not registered with PROSPERO.

#### Inclusion and exclusion criteria

Studies assessing validity and reliability of the FSST in adult populations were sought. The search was restricted to published articles that were carried out after the inception of the FSST in 2002. Studies were excluded if they looked at the FSST in children or defined as anyone under the age of 18, as their outcomes are likely to differ from those in the adult population. Studies that were measuring an intervention using the FSST as an outcome measure were also excluded, as the results would not provide a measure of validity and reliability and prove irrelevant to the review. We did not exclude any papers based on language, as this could exclude potentially relevant articles and a translation service was made available to the reviewers.

### Data extraction and quality assessment

Data extraction forms to measure study quality and risk of bias were taken from the quality assessment of primary Diagnostic Accuracy Studies (QUADAS 2) tool. The QUADAS 2 tool is recommended for use in literature reviews to evaluate the risk of bias and applicability of primary diagnostic accuracy studies [16]. This checklist consists of four key domains: patient selection, index test, reference standard and flow and timing. The reference standards varied in each study due to the multi-factorial nature of balance testing. Therefore, the reviewers chose not to exclude any one reference standard or study on this basis but to compare the FSST to all the stated balance measures for each study.

As the QUADAS 2 tool does not give a measure of reliability, the Consensus-based Standards for the selection of health Measurement Instruments (COSMIN) checklist was used for a measure of reliability. The COSMIN checklist contains standards for evaluating the methodological quality of studies on the measurement properties of health measurement instruments [17]. The checklist was chosen because it is a well recognised and reliable assessment of health measurement instruments which was developed through a multidisciplinary, international Delphi study and included experts from all over the world [17].The checklist looks at nine different domains although only the reliability component was selected for this review.

Two reviewers (MM and KB) independently extracted the data. KB was masked to the key details of each paper. We resolved initial disagreements regarding study quality by discussion until a consensus was reached. Major disagreement was rare, with more minor “low” to “unclear” or “high” to “unclear” being discussed until 100% agreement was reached.

In this review, studies were considered to be of “moderate” quality if they rated low in the QUADAS 2 tool for all domains in both risk of bias and applicability. For reliability, a methodological quality score is determined by taking the lowest rating of any item in a box or a “worst score counts” system [16]. This is because the COSMIN rating score considers a poor score on any item to represent a fatal floor.

To interpret the statistical results of the studies, recognised guideline scores for the strength of the findings were used. For Pearson correlation coefficients (*r*), scores of 0.1 to 0.3 were considered weak, 0.4 to 0.6 were moderate and 0.7 to 0.9 are strong [18]. For Intraclass Correlation Coefficients (ICCs), scores of less than 0.4 were considered poor, 0.4 to 0.59 fair, 0.6 to 0.74 good and 0.75 to 1.00 excellent [19].

To optimise the quality of this report, the PRISMA guidelines and checklist were used during the write-up of this systematic review.

## Results

This section will provide an overall summary of the findings from the review including each studies’ methodological quality score from the QUADAS 2 tool and COSMIN checklist. The validity, reliability and feasibility of each study is also analysed and reported on.

### Study selection

**Fig. 2:**
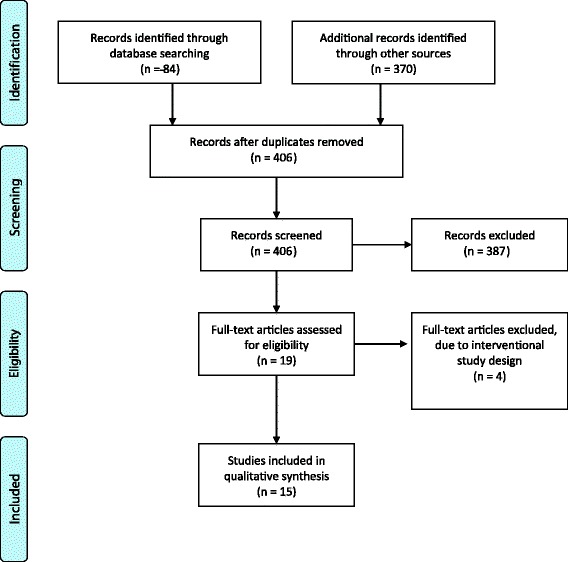
PRISMA flow diagram of selection process

The researchers independently screened all potential papers. Initially, 405 records were identified and a final 15 studies were included in the review. The details of the screening process can be found in the PRISMA flow diagram (Fig. 2).

A summary of the final 15 papers chosen to review can be found in Additional file 1.

### Study bias

The application and conditions varied between studies and variations included changing the canes used to set up the test [20, 21], not allowing participants to use walking aids [21], carrying out the test on a square board [22] and altering the wording of the test to ensure participants were safe throughout the test [23, 24]. One of the studies aimed to validate a modified FSST, in which the sticks were replaced for tape and the participants marked with a fail if they turned to face the stick to step [25]. Additionally, four studies failed to account for order effects of the testing sequence [21, 24, 26, 27], with one study also omitting the rest period given to participants between tests to overcome the effects of fatigue [24].

Whilst assessing reliability, some of the studies did not mention whether raters were blinded to each other’s scores [20, 25, 26]. Varying levels of experience were also described for the raters carrying out the assessments which could allow the test to be carried out by a range of clinicians and researchers alike improving the flexibility of using the measure. Two studies [28, 29] failed to mention the level of experience for the raters making it difficult to generalise the reliability of their results for clinicians.

The majority of studies chose to recruit participants from local community groups, clinics and nursing homes or through advertisement in local papers and flyers [2, 20–25, 27, 28, 30–32]. A selection bias was possible in those studies who recruited participants already taking part in another study [26, 29] and when a consecutive or random sample of participants was not obtained [21, 23].

Sample size varied significantly between studies and depended on the testing population. The larger sample sizes were seen in those patients with multiple sclerosis [32], whilst the smallest included only 20 participants with Huntington’s disease [29]. For reliability, only two studies [21, 28] included the overall number of participants recruited in the analysis. This again reduces the power of the results affecting the overall study quality.

### Study quality

All 15 studies were assessed for methodological quality. For the QUADAS-2 tool (see Table 2), three studies were of moderate quality scoring low in both risk of bias and applicability for all domains [2, 20, 25]. One study rated as having high risk of bias [33], with the other ten rated as having unclear risk of bias [21–24, 26–32].

**Table 2:** QUADAS-2 data extraction score

Studies	Domain 1 patient selection, bias/applicability	Domain 2 index test, bias/applicability	Domain 3 reference standard bias/applicability	Domain 4 flow and timing bias
Dite and Temple [2]	Low/low	Low/low	Low/low	Low
Ibrahim, Altug and Cavlak [28]	Unclear/low	Low/low	Low/low	Low
Blennerhassett and Jayalath [23]	Low/low	Unclear/low	Low/low	High
Goh et al. [26]	Low/low	Unclear/low	Unclear/low	Unclear
Roos et al. [25]	Low/low	Low/low	Low/low	Low
Duncan and Earhart [20]	Unclear/high	Low/low	Low/low	Unclear
McKee and Hackney [24]	Low/low	Unclear/low	Unclear/low	Unclear
Quinn et al. [31]	Low/low	Unclear/low	Unclear/low	Low
Kloos et al. [29]	Unclear/low	Low/low	Low/low	Low
Wagner et al. [27]	Unclear/low	Unclear/low	Low/low	Low
Kalron and Givon [32]	Unclear/low	Unclear/low	Unclear/low	Low
Whitney et al. [21]	Unclear/low	Unclear/low	Unclear/low	Unclear
Choi et al. [33]	Low/low	Low/low	Low/low	Low
Dite et al. [30]	Low/low	Unclear/low	Unclear/low	Low
Schumacher et al. [22]	Unclear/unclear	Unclear/low	Unclear/low	Unclear

For applicability of the study to the research question, a further ten of the studies rated as low concern [21, 23, 24, 27–33], with one study considered to be of high concern [20] and a further study with unclear applicability [22]. Those studies who scored high or unclear in risk of bias failed to clearly state how the index test was conducted or whether participants who were lost to follow up were included in the cohort. Many studies also failed to note whether the index test (FSST) was completed without knowledge of the reference standard tests that had previously been validated in that population.

No study gained an overall “excellent” or “good” score using the COSMIN checklist. It was clear that many of the studies failed to recruit an adequate sample size or did not consider the time interval between the measurements to account for participant fatigue. As a result of this, only three studies gained a “fair” score [2, 21, 31], with the rest scoring “poor” [20, 22–30, 32, 33] (see Additional file 2).

Although all studies were assessed using the COSMIN checklist, three of the studies did not report ICC values nor could the ICC value be calculated from the data presented. Nonetheless, the studies were chosen to be included in the review as they were seen as papers of potential value.

### Validity of the FSST

#### Concurrent validity

Eleven of the 13 studies reviewed measured the concurrent validity of the FSST by measuring it against previously validated tests in the study population [2, 21, 23–29, 32, 33]. Variable correlations were found within each study depending on the reference test used and the study population. Most commonly, the FSST was compared to the timed up and go (TUG) and was shown to have moderate to strong correlations for community-dwelling older adults (*r* = .595–.88, *p* < .001) [2, 28] and in those with balance deficits (*r* = .69, *p* < .01) [21]. Strong correlations for the FSST and TUG were found for Parkinson’s patients (*r* = .73, *p* < .01) [24] and for stroke patients (*r* = .727, *p* = .001) [25].

#### Construct validity

Six studies measured construct validity of the FSST [20, 22, 24, 25, 30, 32]. Two of the studies looked to determine if the FSST could distinguish between Parkinson’s patients who were fallers or non-fallers [20, 24], yet no significant difference was found between the two groups in either of the studies. However, differences between test time for fallers and non-multiple fallers using the FSST was found to be significant for subjects who had undergone unilateral transtibial amputation (*F*_1,35_ = 49.07, *p* < .001) [30] and for those with multiple sclerosis (*F* = 28.3, *p* = .001) [32]. For subjects with knee pain, age related differences were found with the FSST with an odds ratio greater than one (95% CI, OR 1.99; 7.18) meaning those over the age of 65 are more likely to have a slow test time than those under the age of 65, where a slow test time is determined as anything greater than 10 s [22].

#### Predictive validity

A number of studies looked at the validity of the FSST as a predictor for falls in the testing population (see Additional file 3). A range of cut off scores was identified from as low as 9.68 s in those with Parkinson’s disease [20] up to 24 s following unilateral transtibial amputation [30]. Three of the studies were able to identify a significant area under the curve score (range 0.65–0.89), identifying those with dynamic balance impairments using FSST times [20, 21, 26]. However, because of differences in sex, age and body mass index between the subject groups tested, this comparison should be interpreted with caution.

### Reliability of the FSST

#### Inter-rater and intra-rater reliability

Four studies measured inter-rater reliability [2, 25, 26, 28, 33]. Excellent Intraclass Correlation Coefficient (ICC) values were found between physiotherapists carrying out the FSST (ICC = .99) [20, 25]. Good to excellent ICC values were also shown between research assistants carrying out the test (ICC = .86–.99) [2, 26, 33]. Only three studies assessed intra-rater reliability for the FSST, with one showing an excellent level of reliability within raters (ICC = .99) [25] and the other two reporting a good level of reliability within raters (ICC = .83) [26, 33].

#### Test-retest reliability

Ten of the 15 studies reviewed measured test-retest reliability for the FSST. Good to excellent reliability was shown for two separate test scores (ICC = .73–.98) [2, 20, 21, 23–25, 27–29, 31]. One study measured test-retest during three different trials and found from time point one to two the ICC was less (ICC = .735) than from time point two to three (ICC = . 876), and only a moderate correlation was found between test scores at time points one and three (ICC = .543) [24].

### Feasibility

As earlier mentioned, the FSST imposes a testing limit meaning if the participant is unable to carry out each trial twice they are not given a score. This may mean the test is not feasible in those participants who have a significant balance or cognitive impairment as they will find the test too difficult to carry and so the range of possible values will decrease. This was seen in those subjects post stroke with a floor effect of 20% in one study [26] up to 62% of participants having unsuccessful trials at least once in another [23]. One study looking at those patients after stroke overcame this by modifying the FSST to replace the sticks with tape. They found that the proportion of subjects able to complete the modified FSST was significantly greater than those able to complete the FSST (*p =* .04) [25]. Furthermore, studies into the FSST in multiple sclerosis found no floor or ceiling effects whilst carrying out the test, but floor effects were present in the other tests conducted such as the Berg Balance Scale [27].

## Discussion

### Principle findings

The results suggest that the FSST has strong construct and concurrent validity with further analysis needing to be conducted for predictive validity before it can confidently be concluded the FSST is a good predictor of falls. The intra-rater, inter-rater and test-retest reliability within each testing population also produced some encouraging ICC values. Furthermore, the test is relatively inexpensive and quick to administer.

On appraisal of the methodological quality of these studies, however, there was some concern that no study had been able to gain above a fair score for reliability for all of the conditions reviewed. In four of the studies, the time intervals stated for test-retest reliability were also less than the recognised 7-day interval typically found. Therefore, an accurate representation of the stability of FSST performance has not been gauged for the two separate occasions. Interestingly, the rest periods for inter-rater reliability were also variable and may have failed to account for the effects of fatigue. However, it is accepted that fatigue is a subjective concept and for many of the populations within the review the FSST would have been of low physical demand which would not have affected test times. Additionally, only two studies had achieved a moderate quality score for risk of bias and applicability in people post stroke and community-dwelling older adults whilst measuring validity. Poor methodological quality makes it difficult to trust the results of the FSST for all conditions meaning that the quality of the instrument remains unclear [15].

### Review in context with current literature

Since its inception in 2002, the FSST has been validated in a variety of populations with a number showing its ability to highlight falls risk and measure dynamic balance. However, its wide scale use in clinical and rehabilitation settings has not yet been championed. More common well-established measures of balance like the TUG are often favoured by clinicians. Yet the results gleamed from this review for the validity and reliability of the FSST are much the same as the TUG, which has been shown to have excellent concurrent validity when compared with a number of different outcome measures in community-dwelling older adults [34] and in stroke patients [35]. Furthermore, excellent test-retest reliability is shown for the TUG in those with osteoarthritis [36] and stroke [35].

Our review contained papers detailing the use of the FSST across a range of clinical conditions from stroke, to arthritis, and in community-dwelling adults. Of the 15 papers included in the review, two referred to community-dwelling adults without any clinical diagnosis [2, 28], three related to musculoskeletal conditions [22, 30, 32], three to stroke [23, 25, 26], and two each in Parkinson’s disease [20, 24], Huntington’s chorea [29, 31] and multiple sclerosis [27, 33], with one on vestibular deficit [21].

The papers that report the use of FSST in musculoskeletal conditions such as arthritis and amputation were comparable with other measures such as the TUG, offering a different and functionally relevant activity that was useful in identifying falls risk. In patients with knee problems, an association between performance of the FSST and age was found, whilst in hip osteoarthritis, the FSST was found to be reliable between raters in a single session but performed worse than the step test on test retest. Overall, the utility of the FSST in musculoskeletal populations was supported with adequate validity and reliability.

In papers that looked at neurological populations, the results were more variable. Some authors reported that the FSST was a feasible and valid test of dynamic standing balance that was sensitive to change in stroke rehabilitation [23, 26]. However, it has been reported that individuals with stroke had difficulty completing the FSST with problems clearing the walking stick or were unable to complete the test [23, 25], leading to one author developing a modified version of the test using tape rather than sticks to remove the need to step over an obstacle [25]. The difference in ability to complete the unmodified FSST in the stroke population rests with the level of functional impairment of the patients tested. In other neurological populations such as multiple sclerosis, Parkinson’s disease and Huntington’s, the FSST was reported to be a valid and reliable measure in patients with MS and minimal to moderate clinical disability [27]. For Parkinson’s disease, the test was reported to be valid and reliable across a range of disease severity [20, 24]. For Huntington’s disease, the degree of disease severity influenced the reliability and validity of the FSST with good reliability in the pre-manifest stage but less so in manifest HD [31]. In the study by Kloos et al. [29], good to excellent test-retest reliability was demonstrated but the population was restricted to participants in the early to middle stages of HD. Overall, there was strong support for the use of the FSST across a range of clinical populations, with some caveats around its use in more clinically advanced neurologically impaired patients.

It has previously been concluded that no single measure of balance should be used to identify those at risk of falling due to the multifactorial nature of balance [37]. With this in mind, the use of the FSST, which provides a unique measure of rapid step taking in multiple directions, could be used in conjunction with the TUG and other outcome measures, which have shown to provide a measure of strength and the ability to turn about a curve. This would help to provide a more clinically meaningful picture of patients balance and aid clinicians to tailor rehabilitation to those aspects that are contributing to poor balance.

### Limitations

There are some limitations in this systematic review. Only 15 studies could be identified for the review with most showing low reliability and an increasing risk of bias for validity. The majority of studies in this review were assessing the FSST in a population with neurological conditions, and it was apparent the test is vastly under researched in those populations with musculoskeletal conditions who are at an increased risk of poor balance and mobility.

Additionally, the QUADAS-2 tool is more frequently used in studies assessing concurrent validity with some of the questions regarding the reference tests being specifically targeted at these study designs. This tool may, therefore, fail to correctly appraise those studies looking at construct and predictive validity. However, for consistency within the review, this tool was found to be the most indicative of risk of bias and is still useful at capturing the studies ability to correctly measure the FSST’s validity.

Due to the heterogeneity of the studies, a meta-analysis of the studies’ results could not be performed. Furthermore, at review level, only two independent researchers were used. Had a third been available to offer input where there were minor discrepancies in quality assessment scores, the potential for bias from the more experienced reviewer may have been reduced.

### Recommendations for future research

Future studies investigating the validity and reliability of the FSST should follow the test protocol as first described and show in detail the procedures used whilst recruiting and assessing participants. Moreover, the timing between testing for reliability and blinding of raters should be carefully considered before the test is conducted. Further enquiry into the validity of the test for musculoskeletal conditions would be beneficial with the knowledge that these participants are more at risk of falls, and currently, there is little research in this area.

## Conclusions

This systematic review offers insight into the validity and reliability of the FSST for community-dwelling older adults, patients with Parkinson’s disease, Huntington’s disease, multiple sclerosis, vestibular disorders, post stroke, following transtibial amputation, knee pain and hip osteoarthritis. The results of these studies show that the FSST may be an effective and valid tool for measuring dynamic balance and mobility which in turn can predict a participants’ falls risk. Overall, however, the quality of the papers reviewed was at best moderate with key factors needing to be addressed before the tool can be confidently used in these specified populations. Clinicians using the FSST to identify falls risk should do so in conjunction with other multi-factorial fall risk screens.

## Additional files

Additional file 1: Summary of reviewed studies. This file provides a summary table of the reviewed studies and their findings. (DOCX 28 kb)

Additional file 2: COSMIN tool data extraction. This file shows the results from the COSMIN tool quality assessment for reliability. (DOCX 22 kb)

Additional file 3: Summary of predictive validity. This file provides a summary of results from those studies assessing predictive validity. (DOCX 30 kb)

## Abbreviations

6MWT: 6 Minute Walk Test
9HPT: 9 Hole Peg Test
ABC: Acitivies-specific Balance Confidence Scale
BBS: Berg Balance Scale
COSMIN: Consensus based Standards for the selection of health Measurement Instruments
DGI: Dynamic Gait Index
EDSS: Expanded Disability Status Scale
FRT: Functional reach test
FSST: Four square step test
LOS: Limits of stability
MDS-UPDRS-III: Movement Disorders Society–Unified Parkinson Disease Rating Scale-III
QUADAS-2: Quality of primary Diagnostic Accuracy Studies
SLST: Single-leg stance test
ST: Step test
TMT: Tinetti mobility test
TUG: Timed Up and Go
