# Supplementary material for: The validity and reliability of the four square step test in different adult populations: a systematic review
Source: Syst Rev. 2017 Sep 11;6:187. doi: 10.1186/s13643-017-0577-5 (PMC5594590; doi:10.1186/s13643-017-0577-5)
Supplement: Supplementary file 1 — Summary of reviewed studies. This file provides a summary table of the reviewed studies and their findings. (DOCX 28 kb) [file 13643_2017_577_MOESM1_ESM.docx]

| **Authors (Year) and Population** | **Psychometric property** | **Subject demographics**  **(F = Female, M= Male)** | **Results** |
| --- | --- | --- | --- |
| Dite and Temple (2002)  81 community dwelling older adults | Concurrent validity  Inter-rater reliability  Test-retest reliability | Mean age fallers (F=14, M=13) =74.00 (SD±5.68y). Mean age non-fallers (F=14, M=13) = 73.78 (SD±6.09y). Age matched controls (F=14, M=13) = 74.14 (SD±6.07y) | Significant correlations between all measures (p<.001). FSST correlated with ST (r=-.83) FRT (r=-.47) and TUG (r=.88)  Excellent test-retest (ICC =.98) and inter-rater reliability (ICC =.99) |
| Ibrahim, Altug and Cavlak (2015)  80 healthy older adults | Concurrent validity  Test-retest reliability | Mean age of participants = 72.69 (SD±5.09 years)  (F =36, M =44) | Correlation between FSST and SLST (r=-.348, p<.01), FRT (r=-232, p=.05), BBS (r=.0641, p=.001) and TUG (r=.595, p= <.001).  Excellent Test-retest reliability (α=.96 p=<.001) |
| Blennerhassett and Jayalath (2008)  37 subjects with stroke | Concurrent Validity  Test-retest reliability | Mean age of participants = 53 (range 23–75)  (F =12, M =25) | Relationship between FSST and ST at initial, 2 and 4 week follow up (ICC=.73-.86, p<.01).  Excellent test-retest reliability for both tests (ICC.94-.99). |
| Goh et al. (2013)  Community dwelling older adults with chronic stroke (n=15) and healthy control adults (n=15) | Concurrent validity  Inter-rater reliability  Intra-rater reliability | Mean age healthy control adults (F =13, M =2) = 57.30 (SD±3.60)  Mean age chronic stroke (F = 4, M =11) = 57.70 (SD±8.20) | Moderate correlation with FSST and TUG (r=.59, p=.02), in LOS backward direction (r=.64, p=.01) and LOS forward direction (r=.59, p=.02). FSST and BBS not significantly correlated.  Excellent Inter-rater (ICC =.99) and Good Intra-rater reliability (ICC =.83) |
| Roos et al. (2016)  71 community dwelling people with stroke | Concurrent Validity  Construct Validity  Test-retest reliability  Inter-rater reliability  Intra-rater reliability | Validity group mean age (F =25, M =30) = 63.3 (range 39.8 – 87.4)  Test-retest reliability group mean age (F =4, M =13) = 62.5 (range 47.6 – 80)  Inter-rater/ Intra-rater reliability group mean age ( F =9, M =17) = 63.3 (range 45.2 – 81.2) | FSST and mFSST highly correlated with each other (rs= 0.954 p=<0.0001), moderate negative correlations between FSST and BBS (rs= -.685 p=.001) and ABC (rs= -.538 p=.001).  Times to complete tests significantly longer for the SI subjects than the LI subjects (p<.001)  Test-retest reliability excellent (ICC = .85)  Inter-rater reliability excellent (ICC = .99)  Intra-rater reliability excellent (ICC = .99) |

**Additional file 1. Summary of reviewed papers**

**Additional file 1. Summary of reviewed papers (Continued)**

| **Authors (Year) and Population** | **Psychometric property** | **Subject demographics**  **(F = Female, M= Male)** | **Results** |
| --- | --- | --- | --- |
| Duncan and Earhart (2013)  53 individuals with Parkinsons disease | Concurrent Validity  Construct Validity  Inter-rater reliability  Test-retest reliability | Off medication group mean age (F=13, M=15) = 70 (SD±7.4)  On medication group mean age (F= 22, M=31) = 68 (SD±8.5) | Moderate correlation for FSST and Mini-BESTest (rs=-.65), 6MWT (rs= -.52), MDS-UPDRS-III (rs= .61),FTSTS (rs=.58) and 9HPT (rs= .65) significance (p<.001).  Significant difference for those on vs. off medication (p<.03).  Excellent inter-rater reliability for those on medication (ICC.99).  Good test-retest reliability in individuals both on (ICC = .78) and off medication (ICC= .90). |
| McKee and Hackney (2014)  34 Individuals with mild-moderate Parkinsons and a further 24 aged matched individuals | Concurrent validity  Test-retest reliability | Parkinson subjects mean age (F=12, M=19) = 69.65 (SD±7.7)  Older adults mean age (F=17, M=7) = 75.75 (SD±5.0) | FSST correlated with TUG (r=.73), BBS (r=-.66), 6MWT (r=-.46), 30 second chair stand (r=-.475) and gait speed tests (r=-5.6) all significant (*p<*0.01)  Cognitive TUG contributes to FSST performance accounting for 75.9% of the variance  Strong correlation trials 1 and 2 (ICC = 0.735(0.512-0.865)) and 2 and 3 (ICC = 0.876 (0.759-0.939) with high significance (*p<*0.001). |
| Quinn et al. (2013)  75 individuals with Huntingtons Disease | Test-retest Reliability  Minimal Detectable Change | Pre- manifest or manifest HD mean age (F =42, M=33) = 52.12 (SD±11.82) | Excellent test-retest reliability (ICC= .91) in pre-manifest HD and good in manifest HD (ICC=.78). Low MDC scores (1.95) in the pre-manifest HD group and high MDC scores (15.27) in manifest HD. |

**Additional file 1. Summary of reviewed papers (Continued)**

| **Authors (Year) and Population** | **Psychometric property** | **Subject demographics**  **(F = Female, M= Male)** | **Results** |
| --- | --- | --- | --- |
| Kloos et al. (2014)  20 subjects with Huntingtons Disease | Concurrent Validity  Test-retest Reliability | Subjects with HD mean age (F=13, M=7) = 50.9 (SD±13.7) | The FSST was moderately correlated with the TMT (r=-.67, p<.01) and ABC (r=-.57, p<.05).  Test-retest reliability for FSST and TMT good over 6 weeks (ICC=≥0.83). |
| Wagner et al. (2013)  25 Subjects with Multiple Sclerosis | Concurrent Validity  Test-retest reliability | Subjects with Multiple Sclerosis mean age (F= 19, M=6) = 41.6 (SD±9.8) | Excellent correlations between FSST and BBS(rs =-.84), DGI (rs =-.81) and ABC (rs =-.78) and moderate correlation for FSST and EDSS (rs =.73) all significant at (p<.001).  Excellent test-retest reliability (ICC=.92) |
| Kalron and Givon (2016)  218 People with Multiple Sclerosis | Construct Validity  Concurrent Validity | People with Multiple Sclerosis mean age (F=33, M=85) = 43.2 (SD±13.5) | Significant differences for the FSST observed between EDSS disability groups in those with very mild, mild and moderate disability (F=16.565 p <.001).  Fallers demonstrated a significantly slower FSST score than non-fallers (F = 28.3 p <.001).  Moderate correlations between FSST and TUG (r= .652) and 2MWT (r= .575). |
| Whitney et al. (2007)  32 subjects with balance deficits | Concurrent Validity  Test-retest reliability | Subjects with balance deficits mean age (F= 17, M= 15) = 63.7 (SD±17.8) | FSST correlated with TUG (r=.69) gait speed (r=.65) and DGI (r=.51) all significant at p=<.01.  Excellent Test-retest reliability (ICC=.93; 95% CI= .86-.96) |

**Additional file 1. Summary of reviewed papers (Continued)**

| **Title and Authors (Year)** | **Psychometric property** | **Subject tested** | **Results** |
| --- | --- | --- | --- |
| Choi et al. (2014)  31 subjects with Hip Osteoarthritis | Inter-rater reliability  Intra-rater reliability | Subjects with Hip Osteoarthritis mean age (F=18, M=12) = 63.3 (SD±5.71) | Good inter-rater reliability for FSST (ICC = .86, lower 1 sided 95% CI = 7.5)  The FSST and ST had sufficiently low measurement error (<10% of test score).  Good intra-rater reliability (ICC =.83) |
| Dite et al. (2007)  47 subjects following unilateral transtibial amputation | Construct Validity | Multiple fallers mean age (F =3, M =10) = 65.23 (SD±11.18)  Non-multiple fallers mean age (F=7, M= 20) = 59.93 (SD±14.28) | Significant differences between fallers and nonmultiple fallers for FSST at two testing timepoints (F_1,35_=49.07, *P*<.001). |
| Schumacher et al. (2006)  188 subjects with knee pain | Construct Validity | Subjects with knee pain age in decades (F= 102, M= 86) =  40-49 years (n=25)  50-59 years (n=58)  60-69 years (n=58)  70-79 years (n= 41)  > 80 years (n= 6) | Median score test time 10.0s, scores >10s considered slow. Those over the age of 65 are more likely (OR 3.78) to have a slow FSST time than those under 65, 95% CI (1.99;7.18) |
| Abbreviations: **FSST** = Four Square Step Test, **mFSST** = Modified Four Square Step Test, **BBS** = Berg Balance Scale, **ST**= Step Test, **SLST** = Single Leg Stance Test, **FRT** = Functional Reach Test, **TUG** = Timed Up and Go, **LOS** = Limits of stability, **6MWT** = 6 Minute Walk Test, **MDS-UPDRS-III** = Movement Disorders Society–Unified Parkinson Disease Rating Scale-III, **9HPT** = 9 Hole Peg Test, **2MWT** = 2 minute walk test, **HD** = Huntington’s Disease, **MDC** = Minimal Detectable Change, **TMT** = Tinetti Mobility Test, **ABC** = Acitivies-specific Balance Confidence Scale, **EDSS** = Expanded Disability Status Scale, **DGI** = Dynamic Gait Index, **EDSS** = Expanded Disability Status Scale, **SI** = Significantly impaired dynamic balance, **LI** = Less impaired dynamic balance and **OR** = Odds Ratio. | | | |
