# Supplementary material for: The validity and reliability of the four square step test in different adult populations: a systematic review
Source: Syst Rev. 2017 Sep 11;6:187. doi: 10.1186/s13643-017-0577-5 (PMC5594590; doi:10.1186/s13643-017-0577-5)
Supplement: Supplementary file 2 — COSMIN tool data extraction. This file shows the results from the COSMIN tool quality assessment for reliability. (DOCX 22 kb) [file 13643_2017_577_MOESM2_ESM.docx]

| **Studies** | **Q1** | **Q2** | **Q3** | **Q4** | **Q5** | **Q6** | **Q7** | **Q8** | **Q9** | **Q10** | **Q11** |
| --- | --- | --- | --- | --- | --- | --- | --- | --- | --- | --- | --- |
| **Dite and Temple (2002)** | N/A | N/A | Fair | Excellent | Excellent | Excellent | Good | Excellent | Good | Excellent | Good |
| **Ibrahim, Altug and Cavlak (2015)** | N/A | N/A | Good | Poor | Fair | Fair | Good | Fair | Good | Fair | Fair |
| **Blennerhassett and Jayalath (2008)** | Excellent | Excellent | Poor | Excellent | Good | Excellent | Poor | Excellent | Good | Fair | Good |
| **Goh et al.**  **(2013)** | Excellent | Excellent | Poor | Poor | Good | Excellent | Good | Poor | Excellent | Fair | Good |
| **Roos et al. (2016)** | N/A | N/A | Poor | Excellent | Good | Excellent | Good | Excellent | Good | Fair | Good |
| **Duncan and Earhart (2013)** | Excellent | Excellent | Poor | Poor | Good | Fair | Good | Poor | Excellent | Fair | Good |
| **McKee and Hackney (2014)** | Excellent | Excellent | Poor | Poor | Good | Fair | Good | Poor | Good | Excellent | Good |
| **Quinn et al. (2013)** | Excellent | Excellent | Good | Excellent | Good | Excellent | Good | Excellent | Good | Fair | Good |
| **Kloos et al. (2014)** | Excellent | Excellent | Poor | Excellent | Good | Excellent | Good | Excellent | Good | Fair | Excellent |

| **Studies** | **Q1** | **Q2** | **Q3** | **Q4** | **Q5** | **Q6** | **Q7** | **Q8** | **Q9** | **Q10** | **Q11** |
| --- | --- | --- | --- | --- | --- | --- | --- | --- | --- | --- | --- |
| **Wagner et al. (2013)** | N/A | N/A | Poor | Excellent | Good | Excellent | Excellent | Excellent | Poor | Excellent | Good |
| **Kalron and Givon (2016)** | N/A | N/A | Excellent | Poor | Poor | Fair | Good | Poor | Good | Fair | Fair |
| **Whitney et al. (2007)** | N/A | N/A | Fair | Excellent | Good | Fair | Good | Fair | Good | Excellent | Excellent |
| **Choi et al. (2014)** | Excellent | Excellent | Poor | Excellent | Excellent | Excellent | Good | Excellent | Excellent | Excellent | Excellent |
| **Dite et al. (2007)** | Excellent | Fair | Fair | Excellent | Good | Fair | Good | Fair | Good | Excellent | Poor |
| **Schumacher et al. (2006)** | Good | Fair | Excellent | Excellent | Good | Fair | Good | Fair | Fair | Fair | Poor |
