# Supplementary material for: The validity and reliability of the four square step test in different adult populations: a systematic review
Source: Syst Rev. 2017 Sep 11;6:187. doi: 10.1186/s13643-017-0577-5 (PMC5594590; doi:10.1186/s13643-017-0577-5)
Supplement: Supplementary file 3 — Summary of predictive validity. This file provides a summary of results from those studies assessing predictive validity. (DOCX 30 kb) [file 13643_2017_577_MOESM3_ESM.docx]

| **Predictive Validity** | | |
| --- | --- | --- |
| **Authors (Year) and Population** | **Study Demographics** | **Results** |
| Dite and Temple (2002)  81 community dwelling older adults | Mean age fallers (F=14, M=13) =74.00 (SD±5.68y). Mean age non-fallers (F=14, M=13) = 73.78 (SD±6.09y). Age matched controls (F=14, M=13) = 74.14 (SD±6.07y) | Multiple fallers = >15 seconds score  Non-multiple fallers = ≤15 seconds.  At 15 seconds, the FSST has a positive predictive value of 86% and a negative predictive value of 94% for the sample tested. |
| Blennerhassett and Jayalath (2008)  37 subjects with stroke | Mean age of participants = 53 (range 23–75)  (F =12, M =25) | Total of 5 fallers. All had unsuccessful trials during the FSST, and 2 could not achieve a FSST score at the initial test. At the initial assessment, the 3 fallers who achieved a FSST score took between 15.6 to 27.7 seconds to complete the test. |
| Goh et al. (2013)  Community dwelling older adults with chronic stroke (n=15) and healthy control adults (n=15) | Mean age healthy control adults (F =13, M =2) = 57.30 (SD±3.60)  Mean age chronic stroke (F = 4, M =11) = 57.70 (SD±8.20) | An FSST cutoff time of 11 seconds was found to differentiate between the healthy controls and subjects with stroke (sensitivity, 73.3%; specificity, 93.3%; AUC=0.867; *P*≤.001). |
| Duncan and Earhart (2013)  53 individuals with Parkinsons disease | Off medication group mean age (F=13, M=15) = 70 (SD±7.4)  On medication group mean age (F= 22, M=31) = 68 (SD±8.5) | FSST AUC=0.65 (95% CI: 0.43-0.80). FSST cut off score =9.68 seconds (sensitivity = .73, specificity = .57). The positive and negative likelihood ratios for those scoring above the cutoff were 1.7 (95% CI: 1.00-2.73) and 0.48 (95% CI: 0.17- 1.27), respectively. The post-test probability of a fall for those with scores above the cutoff was 31%, compared to a 21% pre-test probability. |
| Whitney et al. (2007)  32 subjects with balance deficits | Subjects with balance deficits mean age (F= 17, M= 15) = 63.7 (SD±17.8) | FSST AUC=0.89 (*P*<.01; 95% CI, 0.78–1.00). FSST cut off score = 12 seconds (sensitivity of 80% and specificity of 92%) for the identification of subjects with 1 or more risk factors for falls in this sample of subjects with vestibular disorders. |
| **Authors (Year) and Population** | **Study Demographics** | **Results** |
| Dite et al. (2007)  47 subjects following unilateral transtibial amputation | Multiple fallers mean age (F =3, M =10) = 65.23 (SD±11.18)  Non-multiple fallers mean age (F=7, M= 20) = 59.93 (SD±14.28) | FSST cut off score = 24 seconds (Sensitivity 92%, Specificity 93%) with positive predictive value 86% and negative predictive value 96% for falls. |
| Abbreviations: **FSST** = Four Square Step Test and **AUC =** area under the curve | | |
